# Bacterial adhesion inhibitor prevents infection in a rodent surgical incision model

**DOI:** 10.1080/21505594.2020.1772652

**Published:** 2020-06-03

**Authors:** R. M. Huebinger, D. H. Do, D. L. Carlson, X. Yao, D. H. Stones, M. De Souza Santos, D. P. Vaz, E. Keen, S. E. Wolf, JP Minei, K. P. Francis, K. Orth, A. M. Krachler

**Affiliations:** a Department of Surgery, Division of General and Acute Care Surgery, University of Texas Southwestern Medical Center, Dallas, TX, USA; b School of Biosciences, Institute of Microbiology and Infection, University of Birmingham, Birmingham, UK; c University of Gloucestershire, School of Natural and Social Sciences, Cheltenham, UK; d Department of Molecular Biology, University of Texas Southwestern Medical Center, Dallas, TX, USA; e Department of Microbiology and Molecular Genetics, University of Texas Health Science Center at Houston, McGovern Medical School, Houston, TX, USA; f UTMB Department of Surgery, Shriners Hospitals for Children, Galveston, TX, USA; g Perkin Elmer, Hopkinton, MA, USA; h Department of Biochemistry, University of Texas Southwestern Medical Center, Dallas, TX, USA; i Howard Hughes Medical Institute, University of Texas Southwestern Medical Center, Dallas, TX, USA

**Keywords:** Surgical infection, incision, laceration, bacterial adhesion, anti-virulence, pseudomonas, staphylococcus

## Abstract

Surgical site infection risk continues to increase due to lack of efficacy in current standard of care drugs. New methods to treat or prevent antibiotic-resistant bacterial infections are needed. Multivalent Adhesion Molecules (MAM) are bacterial adhesins required for virulence. We developed a bacterial adhesion inhibitor using recombinant MAM fragment bound to polymer scaffold, mimicking MAM7 display on the bacterial surface. Here, we test MAM7 inhibitor efficacy to prevent Gram-positive and Gram-negative infections. Using a rodent model of surgical infection, incision sites were infected with antibiotic-resistant bioluminescent strains of *Staphylococcus aureus* or *Pseudomonas aeruginosa*. Infections were treated with MAM7 inhibitor or control suspension. Bacterial abundance was quantified for nine days post infection. Inflammatory responses and histology were characterized using fixed tissue sections. MAM7 inhibitor treatment decreased burden of *S. aureus* and *P. aeruginosa* below detection threshold. Bacterial load of groups treated with control were significantly higher than MAM7 inhibitor-treated groups. Treatment with inhibitor reduced colonization of clinically-relevant pathogens in an *in vivo* model of surgical infection. Use of MAM7 inhibitor to block initial adhesion of bacteria to tissue in surgical incisions may reduce infection rates, presenting a strategy to mitigate overuse of antibiotics to prevent surgical site infections.

## Introduction

Surgical site infections (SSIs) have become the most common and costly of hospital-acquired infections. It is estimated that 20% of all hospital-acquired infections are related to surgical site infections, which directly increase hospital length of stay and risk of mortality. Of the in-hospital mortalities with an SSI at the time of death, 77% of cases are directly attributable to the surgical site infection [1,2]. Despite the guidelines currently in place to prevent or reduce the incidence of SSIs, they remain a persistent and costly challenge[3].

With the increase in antibiotic resistant bacteria and the narrowing pipeline for developing new effective antimicrobials, the challenges for treating infection are ever more difficult. ESKAPE pathogens (*Enterococcus faecium, Staphylococcus aureus, Klebsiella pneumoniae, Acinetobacter baumannii, Pseudomonas aeruginosa* and *Enterobacter* species) are frequently associated with hospital acquired infections [4–6] and are the predominant pathogens identified in combat casualties [6–8]. Due to drug efflux mechanisms and genetically acquired drug resistance, antibiotic tolerance has increased in these species[9]. These characteristics have led to the rise of multidrug-resistant isolates that are observed in patients and transferred between patients through healthcare personnel or hospital equipment [6,10,11]. The intrinsic modes of bacterial defense allow for persistence in clinical settings, and, the current treatments that use more antibiotics, are associated with increasing antimicrobial resistance[12]. Given the link between increased antibiotic use and the increases in antibiotic resistance, a large interdisciplinary task force highlighted the need for better stewardship of antimicrobial agents across surgical disciplines to decrease the expansion of antimicrobial resistance in surgical infections[12]. An urgent need is now recognized for new antimicrobial drugs. Bactericidal and bacteriolytic antimicrobials exert strong selective pressure, leading to the rapid emergence of drug-resistance. Thus, antimicrobials that target virulence, rather than growth, are sought out as a potential alternative [13,14]. Such anti-virulence drugs are thought to exert no selective pressure on the emergence of antimicrobial resistance[15]. There are several different strategies that have been explored for their potential to prevent or treat bacterial infections[16]. These have included compounds that target bacterial attachment/adherence [17,18], biofilm formation [19], secretion systems [20,21], or bacterial toxins [22–25]. Often these compounds target a specific bacterial strain or species, and do not have broad-spectrum efficacy[16].

Recently, we identified Multivalent Adhesion Molecules (MAMs) as a widespread family of bacterial adhesins that facilitate bacterial colonization of host tissues[26]. Multivalent adhesion molecule 7 (MAM7) is a bacterial outer membrane protein that facilitates host cell adhesion by Gram-negative bacteria[26]. We engineered a bacterial adhesion inhibitor that consists of a recombinant fragment of MAM7 chemically coupled to a micron-sized, spherical polystyrene scaffold [27] to mimic its endogenous presentation on the bacterial surface. This inhibitor attaches to host surface receptors fibronectin and phosphatidate, which are commonly used by bacteria to colonize tissues, inhibiting bacterial attachment by competitive exclusion[28]. We evaluated the MAM7-based adhesion inhibitor specifically for its efficacy against multidrug-resistant bacteria isolated from wounded military personnel, including *A. baumannii, K. pneumoniae, P. aeruginosa* and ESBL-producing *E. coli*, and found it to be effective against 21 out of 25 (84%) of tested isolates *in vitro*[29]. MAM7 inhibitor beads have been used *in vitro* to prevent Gram-negative pathogens and methicillin resistant *Staphylococcus aureus* from binding to host cells, thereby inhibiting subsequent infection and cytotoxicity in cultured cell models [29–32]. To our knowledge, MAM7 inhibitor beads are the first reported virulence-targeting compound with a broad spectrum activity against both Gram-positive and Gram-negative bacteria. *In vitro* and *in vivo* studies showed that MAM7 inhibitor beads do not interfere with wound healing [30,33]. Recently, we tested the efficacy of MAM7 inhibitor beads using an *in vivo* rodent model of multidrug-resistant *Pseudomonas aeruginosa* burn wound infection[33]. The MAM7-based adhesion inhibitor restricted both bacterial load and spatial spread of the infection in burn wounds[33].

The aim of this current study was to test if MAM7 inhibitor beads could prevent infections in a clinically relevant model of surgical site infection. In this model, we used two species of drug-resistant bacteria that are frequently associated with surgical site infections, *Staphylococcus aureus* and *Pseudomonas aeruginosa*.

## Materials and methods

### Bacterial strains and growth conditions

*Pseudomonas aeruginosa* strain Xen5 (PerkinElmer), a bioluminescent derivative of a blood isolate from a septic patient (strain ATCC 19660), was used throughout the experiments. The strain is resistant to carbenicillin, chloramphenicol, tetracycline and trimethoprim by Kirby-Bauer Disk Diffusion Test. *Staphylococcus aureus* strain Xen36 (PerkinElmer), a bioluminescent derivative of a clinical isolate from a bacteremic patient (ATCC 49525). The strain is resistant to kanamycin and penicillin by Kirby-Bauer Disk Diffusion Test. *P. aeruginosa* was grown in Luria-Bertani broth (LB) and *S. aureus* was grown in Trypticase Soy Broth (TSB) at 37°C under constant aeration. Inoculum bacteria was grown in log-phase to an optical density of 1.0. Bacteria suspended in LB or TSB media was introduced directly into the wound via pipette.

### Synthesis of inhibitor and control beads

MAM7 (VP1611)ΔN44 was expressed with an N-terminal GST-tag, and purified as previously described[26]. Purified GST-MAM7ΔN44 or GST alone were chemically coupled to polystyrene microbeads, as previously described [27], to produce GST-MAM7 beads (inhibitor) and GST control beads, respectively.

### Animal protocols, incision infection model

All animal protocols were reviewed and approved by the University of Texas Southwestern Medical Center Institutional Animal Care and Use Committee and followed the ARRIVE guidelines for animal research[34]. Adult male Sprague-Dawley rats (276–300 g) were obtained from Envigo Laboratories (Houston, TX). Animals were acclimated in house for seven days after arrival at UTSW. During acclimation rats had access to *ad libitum* commercial rat chow and water. Rats were housed under a 12 hour light/dark cycle. After acclimation, rats were individually housed for the remainder of the experiment. The surgical infection model described by Rupp et al [35]. and Kuklin et al [36]. were used with the following modifications. Rats were deeply anesthetized with isoflurane. While under anesthesia, the surgical area was shaved. With a scalpel, a 4 to 5 cm incision was constructed parallel to the vertebral column with a sterile scalpel. The incision was approximately 1 cm in depth into the paraspinous muscle. 7.5⋅10^7^ CFU of *P. aeruginosa* Xen5 or 1⋅10^7^ CFU of *S. aureus* Xen36 were directly applied into the incisional wound, followed by application of suspensions containing 3⋅10^8^ GST-MAM7 beads or GST control beads. The 10^7^ CFU dose was previously tested in this model producing a consistently reproducible infection (unpublished data). A total of 10 individual animals were used for each condition (i.e. n = 10 *P. aeruginosa* + MAM beads; n = 10 *P. aeruginosa* + Control beads; n = 10 *S. aureus* + MAM beads; n = 10 *S. aureus* + Control beads). Group size for animals inoculated with only bacteria was six. Group size was determined based upon previously published data[33]. Subsequently the incision was sutured closed immediately with 3–0 Prolene suture. Sutures remained in the animal for the duration of the experiment. Animals were imaged daily, using an IVIS Spectrum In vivo Imaging System (PerkinElmer). Measurements of photon flux emitted from bioluminescent bacteria in the wound were used as a proxy for bacterial abundance/burden. Photon flux has been used previously to determine bacterial abundance, and correlates with colony forming unit data [37,38]. Animals were sacrificed if they became moribund, or at the end of the experiment (9 days after surgery). At the end of the experiment, animals were euthanized via exsanguination by cardiac puncture while under isoflurane anesthesia. Blood and tissue were collected from each animal immediately postmortem. All procedures (surgical, imaging, and euthanasia) were conducted in the morning during the light cycle. Daily mean bacterial burdens ± standard errors of the mean (sem) were calculated for each experimental group. Statistical significance in bacterial burdens between groups compared to untreated, infected animals was then determined by analysis of variance (ANOVA) and Dunnett’s multiple comparisons test, using GraphPad Prism 7. Adjusted P-values of P˂0.0001 (****) indicate extremely significant, P˂0.05 (*) significant, and P ≥ 0.05 (ns) not significant results, respectively.

### Tissue preparation and histology

A portion of the dorsal skin was fixed overnight in 10% neutral buffered formalin (NBF) and paraffin embedded. Tissue sections (5 μm) were deparaffinized and rehydrated using graded alcohol concentrations followed by H&E staining or immunofluorescence (IF) staining. For IF staining, formalin-fixed and paraffin embedded skin tissue sections were deparaffinized in xylene and rehydrated using graded alcohol concentrations. Samples were then boiled in 10 mM citric acid for 20 min to retrieve epitopes. Sections were permeabilized with 1% Triton X-100 and blocked with 2% BSA in PBS-T. Primary antibody incubation (anti-myeloperoxidase, 1:100, Abcam ab9535) was carried out overnight at 4°C in PBS-T + 1% BSA, and secondary antibody incubation (anti-rabbit Alexa-488, 1:500, ThermoFisher A-11034) was performed over 1 h at room temperature. Sections were finally stained for DNA using Hoechst (ThermoFisher, 33342) and mounted (Prolong gold antifade, ThermoFisher P36930). Fluorescence images were acquired on a Zeiss LSM710 confocal microscope and processed using Image J and CorelDraw X5. H&E images were examined for gross morphology and IF images were examined for gross presence/abundance of neutrophils.

### Wound healing RT-qPCR assays

In an effort to examine for potential inhibitory effects that MAM7 may have on wound healing, a group of non-infected animals received sham treatment (N = 3), control beads (N = 3), or MAM7 inhibitor (N = 3) within the surgical incision. Animals that were sham treated received a surgical incision and then were sutured closed. Additionally, we measured expression levels of matrix metalloproteinases (matrix metalloproteinase 2 (MMP-2), matrix metalloproteinase 9 (MMP-9) and tissue inhibitor of metallopeptidase (TIMP-1)) to determine the effects on wound healing by real-time quantitative PCR (RT-qPCR). RNA was extracted from a portion of dorsal skin at the incision site using the RNAeasy Universal kit (Qiagen). cDNA conversion was conducted using the QuantiTect reverse transcription kit according to the manufacturer’s protocol (Qiagen). Quantitative assessment of each gene transcript was conducted in triplicate for each individual animal with the TaqMan Fast Advanced Master Mix and TaqMan probes specific for each gene according to the manufacturer’s protocol (PerkinElmer). Levels of gene expression were calculated using the ΔΔCt method normalized to GAPDH (Glyceraldehyde 3-phosphate dehydrogenase) expression levels. The mean normalized ΔΔCt values for each group were compared using paired t-tests in GraphPad Prism 7. P-values<0.05 were interpreted as statistically significant.

### Bacterial attachment to microbeads

GST was chemically coupled to polymer beads as described above. To allow visualization of beads by fluorescence microscopy, beads with an identical surface chemistry as those above, but with a fluorescent blue core were used (Sigma, #L0280). *P. aeruginosa* Xen5 and *S. aureus* Xen36 were grown at 37°C for 16 h as described above. Bacteria were washed with DMEM, and added to GST coupled beads in DMEM to give final concentrations of 5.6 ⋅10^8^ CFU/ml of *P. aeruginosa* + 1.2 ⋅10^9^ beads/ml, and 4⋅10^7^ CFU/ml of *S. aureus* + 1.2 ⋅10^9^ beads/ml, respectively, reflecting the initial ratios of bacterial inocula and beads used in the laceration model. Suspensions of bacteria and beads were incubated at 37°C under static conditions. At 24 and 48 h, samples were removed and bacteria stained using the membrane dye FM 4–64FX according to the manufacturer’s protocol. Samples were mounted on 1.5% agarose pads and imaged using an Olympus IX83 inverted microscope fitted with a FV3000 laser scanning confocal system and a Plan Super Apochromat 100 × 1.40NA oil objective. Images were processed using Olympus CellSens Dimension software and deconvolution package (5 iterations). For each field, z-stacks were collected (0.2 µm increments) and representative maximum intensity projections are shown.

## Results

**Figure 1.**
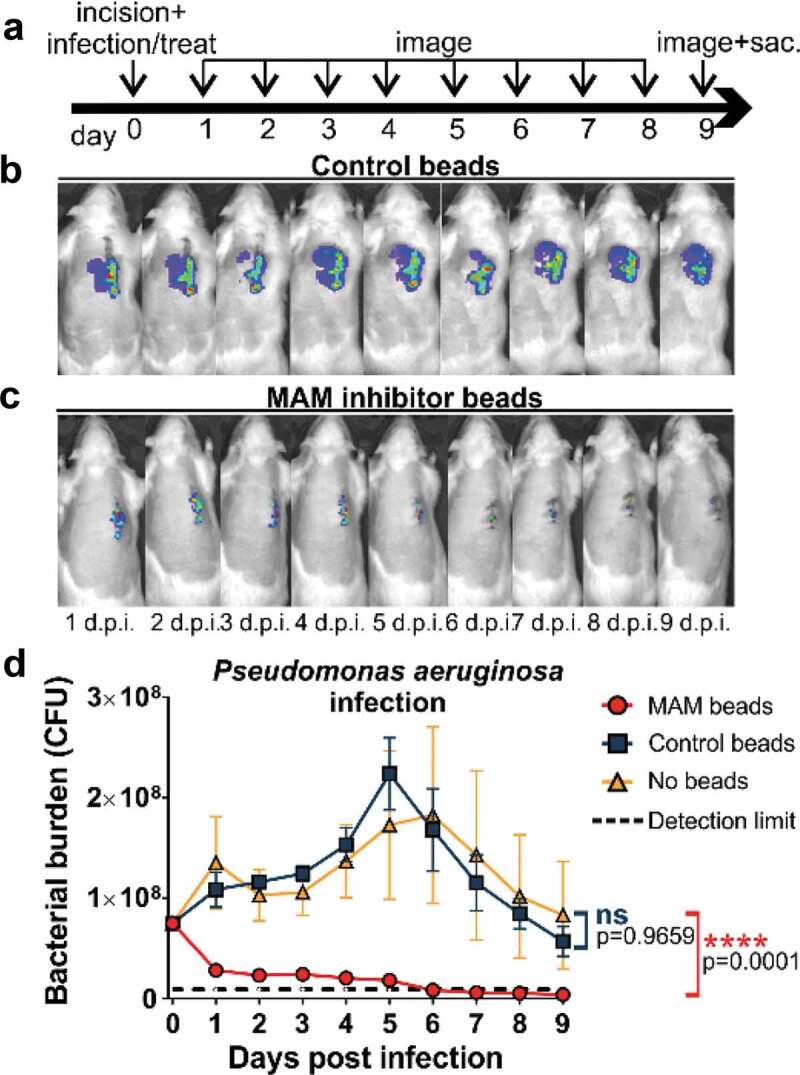
Treatment of surgical laceration wounds with a MAM7 inhibitor decreases *Pseudomonas aeruginosa* bacterial burden and spatially constrains the spread of infection. (a) Timeline of surgery, infection, inhibitor dosing, and imaging regimen. On day 0, animals were infected with 7.5⋅10^7^ CFU of *P. aeruginosa*, and treated with 3⋅10^8^ GST-MAM7 beads (inhibitor) or GST control beads, or the infection left untreated (no beads). Representative daily bioluminescence images of infected surgical incisions treated with GST control beads (b) or GST-MAM7 inhibitor beads (c). (d) Quantification of *P. aeruginosa* bacterial burden (as colony forming units, CFU) in animals treated with GST-MAM7 inhibitor beads (red), GST control beads (blue), and infected but otherwise untreated animals (orange), based on IVIS biophotonic imaging. Dotted black line represents detection limit (9⋅10^5^ CFU). Values are mean burdens ± SEM (n = 10 for MAM bead- and control bead treatment groups; n = 6 for untreated infection group). Statistical significance between burdens in untreated infections relative to MAM inhibitor bead treatment and control bead treatment was determined by one-way ANOVA and a Dunnett’s multiple comparisons test. Adjusted P-Values are indicated in the graph.

We explored the efficacy of MAM7 bacterial adhesion inhibitor against bacterial infections of *Pseudomonas aeruginosa* and *Staphylococcus aureus* in an *in vivo* model of surgical site infection. After creation of the surgical incision, animals were inoculated with 7.5⋅10^7^ CFU of *Pseudomonas aeruginosa* or 1⋅10^7^ CFU of *Staphylococcus aureus*. After inoculation, a suspension of adhesion inhibitor (3⋅10^8^ GST-MAM7-coupled beads) or control beads (3⋅10^8^ GST-coupled beads were applied directly into the surgical site, and incisions were sutured closed (Day 0, Figure 1(a)). Daily quantification of bacterial abundance was carried out by IVIS imaging (Figure 1(a)), and photon flux data was converted into bacterial burden by measuring the flux of known CFUs for each bacterial strain (see methods section). The *P. aeruginosa* burden in the MAM7 inhibitor treated group consistently decreased over the experimental time course (Figure 1(c)), resulting in a 10-fold reduction in the GST-MAM7 bead treated group relative to the control group within the first 24 hours (Figure 1(d)). Bacterial burden in the MAM7 inhibitor treatment group further declined between days 1–9 post surgery, and was as low as the detection limit at 5–9 days. In the presence of control beads, the inoculum of *P. aeruginosa* introduced into the incision doubled within the initial 24 hours following surgery and steadily increased thereafter, reaching a maximum at day 5 post infection (Figure 1(b,d)). Between day 5–9, the experimental endpoint, the bacterial burden decreased back to the original inoculum, but was not resolved to the extent seen in animals treated with MAM7 inhibitor beads (Figure 1(b-d)). Bacterial burden in animals inoculated with bacteria, but left untreated (no MAM7 adhesion inhibitor or control beads) showed a similar profile to those treated with GST control beads (Figure 1(d)).

**Figure 2.**
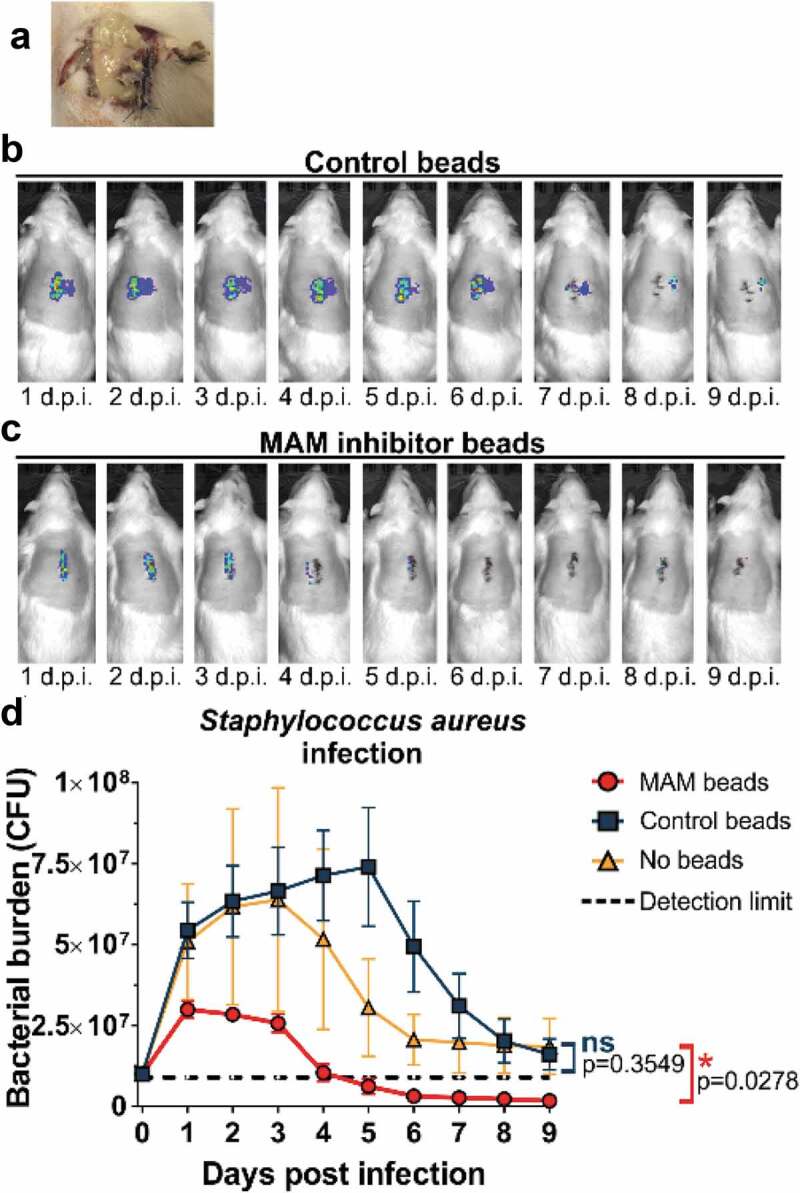
Treatment of surgical laceration wounds with a MAM7 inhibitor decreases *Staphylococcus aureus* bacterial burden and spatially constrains the spread of infection. On day 0, animals were infected with 10^7^ CFU of *S. aureus*, and treated with 3⋅10^8^ GST-MAM7 beads (inhibitor) or GST control beads, or the infection left untreated (no beads). (a) Example of an abscess formed in an infected laceration treated with control beads. Representative daily bioluminescence images of infected surgical incisions treated with GST control beads (b) or GST-MAM7 inhibitor beads (c). (d) Quantification of *S. aureus* bacterial burden (as colony forming units, CFU) in animals treated with GST-MAM7 inhibitor beads (red), GST control beads (blue), and infected but otherwise untreated animals (orange), based on IVIS biophotonic imaging. Dotted black line represents detection limit (9⋅10^5^ CFU). Values are mean burdens ± SEM (n = 10 for MAM bead- and control bead treatment groups; n = 6 for untreated infection group). Statistical significance between burdens in untreated infections relative to MAM inhibitor bead treatment and control bead treatment was determined by one-way ANOVA and a Dunnett’s multiple comparisons test. Adjusted P-Values are indicated in the graph.

Our previous *in vitro* studies showed that GST-MAM7 beads could block host cell colonization by Gram-positive bacteria that share common surface receptors with MAM7, in particular by *S. aureus*, which also attach to fibronectin to colonize host tissues[29]. These prior studies suggested that GST-MAM7 beads may potentially be able to inhibit *S. aureus* tissue colonization *in vivo*. Here, we tested this hypothesis by determining the *in vivo* efficacy of GST-MAM7 beads against *S. aureus* infection in the surgical site infection model. Incisions infected with *S. aureus* and treated with GST control beads did not fully heal, and in many cases animals developed bacterial abscesses under the skin (Figure 2(a)). In contrast, infected incisions treated with GST-MAM7 beads had visually resolved the infection by 9 days post-surgery, showed minimal bacterial infiltration, and the wound was fully healed. IVIS imaging data was in line with visual inspection of surgical sites: In incisions treated with GST control beads, *S. aureus* rapidly expanded (approx. 5-fold within the initial 24 hours post inoculation), and the bacterial burden peaked at 5 days post infection (Figure 2(b,d)), similar to what was observed with *P. aeruginosa* (Figure 1). In contrast, bacterial load remained restricted in GST-MAM7 treated incisions (peak load approx. 3-fold lower compared to GST control group, Figure 2(c,d)), and steadily decreased thereafter, dropping below detectable burdens at 4 days post-surgery (Figure 2(d)). Bacterial abundance in animals inoculated with *S. aureus* but left untreated (no MAM7 adhesion inhibitor or control beads) showed a similar profile as in those treated with GST control beads (Figure 2(d)).

**Figure 3.**
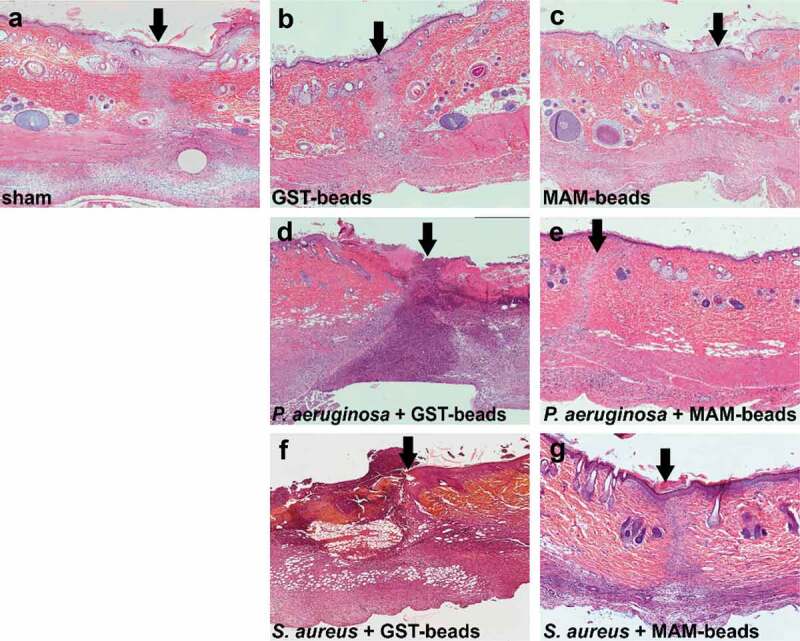
Histology of fixed skin sections from surgical incision model. Skin sections from tissues at 9 days post incision were subjected to hematoxylin and eosin staining. Dorsal skin sections are oriented with the epidermis at the top, and arrows denote the incisional site. Sections of uninfected incisions that were sham treated (a), treated with GST control beads (b), or GST-MAM7 inhibitor beads (c), or sections from incisions that were infected with 7.5 × 10^7^ CFU of *Pseudomonas aeruginosa* and treated with GST control beads (d) or GST-MAM7 inhibitor beads (e), or infected with 10^7^ CFU of *Staphylococcus aureus* and treated with GST control beads (f) or GST-MAM7 inhibitor beads (g) are shown.

Fixed sections taken from surgical incisions were subjected to H&E staining (Figure 3) and examined histologically. Uninfected incisions treated with GST control beads (Figure 3(b)) or GST-MAM7 inhibitor beads (Figure 3(c)) did not show any signs of disrupted wound healing, and were histologically similar to the sham treatment incisions (Figure 3(a)). Within the infected groups, incisions infected with *P. aeruginosa* and *S. aureus* and treated with control beads (Figure 3(d,f)) showed extensive tissue damage and immune cell infiltration, while incisions infected with either *P. aeruginosa* or *S. aureus* and treated with MAM7 inhibitor beads (Figure 3(e,g)) histologically resembled the sham treatment incisions at 9 days post-surgery (Figure 3(a)). The epidermis in groups treated with MAM7 inhibitor beads was fully healed at 9 days post-surgery. Both the *P. aeruginosa* and *S. aureus* infections treated with GST control beads failed to fully restore the epidermis at 9 days post-surgery. Uninfected groups treated with MAM7 inhibitor beads or GST control beads were able to restore the integrity of the epidermis at 9 days post-surgery (Figure 3(b,c)).

In an effort to quantify the effects of GST-MAM7 or GST control beads on wound healing, expression levels of several matrix metalloproteinases were measured at the wound site 9 days post-surgery. The expression levels of matrix metalloproteinases at the wound site were measured by quantitative PCR in the uninfected incision groups only. Levels of MMP-2 (2.64 ± 0.16: 2.74 ± 0.16: 3.1 ± 0.6, p > 0.57), MMP-9 (3.81 ± 0.63: 5.18 ± 1.1: 4.86 ± 0.76, p > 0.38), and TIMP-1 (3.05 ± 0.73: 3.4 ± 0.72: 2.42 ± 0.39, p > 0.26) were not significantly different between sham treated incisions, and incisions treated with GST-MAM7 beads, or GST control beads.

**Figure 4.**
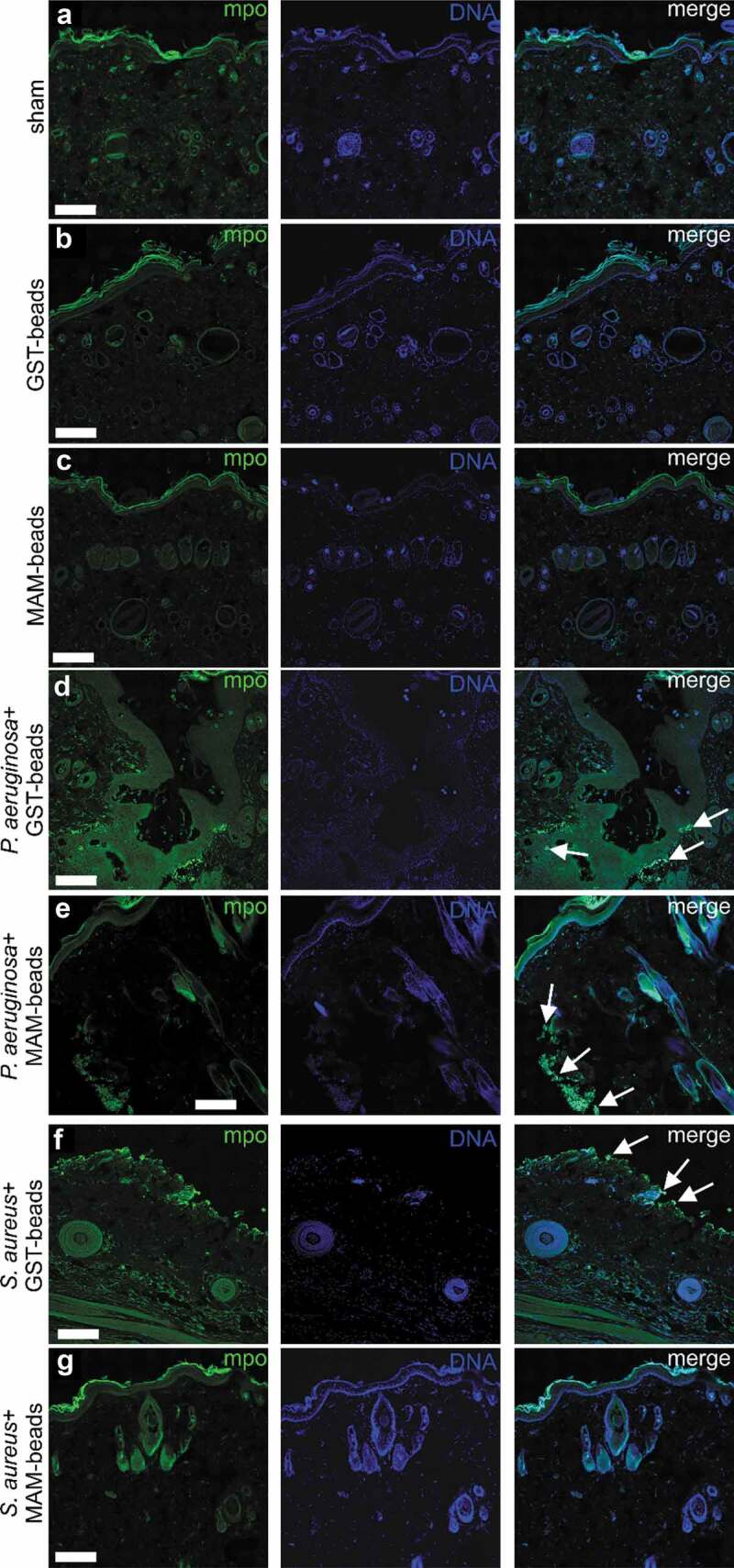
Neutrophil infiltration of surgical incisions. Immunofluorescence images of dorsal skin sections at 9 days post incision. Sections were stained with anti-myeloperoxidase (mpo) and anti-rabbit Alexa-488 to visualize neutrophils (green), and with Hoechst 33342 to visualize DNA (blue). Scale bars, 200 μm; Arrows mark examples of neutrophil clusters. Imaged incisions were either uninfected and sham treated (a), treated with GST control beads (b), or GST-MAM7 inhibitor beads (c), or infected with of *Pseudomonas aeruginosa* and treated with GST control beads (d), or GST-MAM7 inhibitor beads (e), or infected *Staphylococcus aureus* and treated with GST control beads (f) or GST-MAM7 inhibitor beads (g).

Infiltration of neutrophils was examined by myeloperoxidase staining (Figure 4). An absence of neutrophils was noted in the sham (Figure 4(a)) and in the uninfected lacerations treated with GST control beads (Figure 4(b)) or GST-MAM7 inhibitor beads (Figure 4(c)). The presence of neutrophils was observed in both *P. aeruginosa* and *S. aureus* infected lacerations treated with GST control beads (Figure 4(d,f)). Additionally, neutrophils were detected at the site of infection in lacerations infected with *P. aeruginosa* and treated with GST-MAM7 beads (Figure 4(e)). Within *S. aureus* infected incisions treated with GST-MAM7 beads, neutrophils were notably absent (Figure 4(g)).

**Figure 5.**
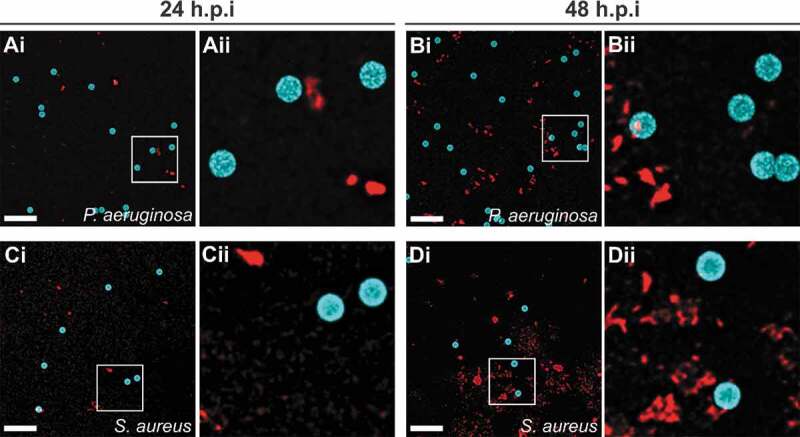
Bacteria do not adhere to functionalized microbeads used to treat infected lacerations. *P. aeruginosa* (a, b) or *S. aureus* (c, d) were incubated with GST control beads for 24 h (a, c) or 48 h (b, d) and imaged. Representative maximum intensity projections of fluorescence confocal z-stack images are shown. Microbeads (blue); Bacteria (FM 4–64FX membrane stain, red); Aii-Dii represent magnified sections as denoted by white squares in Ai-Di. Scale bars, 10 µm.

Attachment of bacteria to GST-functionalized microbeads was examined following co-incubation of beads and *S. aureus* or *P. aeruginosa* in DMEM (Figure 5). An absence of bacterial attachment to the microbeads was noted at both 24 (Figure 5(a,c)) and 48 hours of incubation (Figure 5(b,d)), for both *S. aureus* and *P. aeruginosa*.

## Discussion

Collectively our data demonstrates the effectiveness of a virulence targeting antimicrobial compound with broad specificity in an *in vivo* model of surgical infection. Surgical infections remain a persistent problem and are the major complication following surgical intervention. Previously we reported that microbeads functionalized with a recombinant fragment of the adhesin MAM7 (MAM7-beads) could prevent *Pseudomonas aeruginosa* colonization and spread of infection in a burn wound model[33]. Although *Pseudomonas* has a high prevalence in burn wound infections [7,39], surgical infections are caused by a broader array of pathogens. This includes both Gram-negative and Gram-positive organisms, like *Staphylococcus aureus*. Although previous *in vitro* tests have demonstrated the efficacy of a MAM7 based inhibitor against both Gram-negative and Gram-positive pathogens, it had not been tested in an *in vivo* model of infection against Gram-positive pathogens.

Here, we tested the ability of a MAM7 inhibitor against isolates of both a Gram-negative and a Gram-positive organism that exhibited broad resistance to antibiotics. The typical clinical threshold for determining a clinically significant infection is >10^5^ colony forming units [40,41]. In an effort to exceed this threshold and mimic a high risk of surgical site infection, we used a dose of ≥10^7^ CFU of each bacterial species to infect the surgical site. The 10^7^ CFU dose was previously tested in this model producing a consistently reproducible infection (unpublished data). Subsequent to the incision, the infection was treated with an excess of MAM7 inhibitor or control beads and was then sutured closed. At 24 hours after surgery, there was a noticeable and statistically significant difference in bacterial abundance between the MAM7 and control bead treated groups for both the *S. aureus* and *P. aeruginosa* infections. Based upon previous *in vitro* data, the MAM7 inhibitor prevents the bacterial species from attaching and allows for normal clearance by the hosts immune system. This treatment allowed for the immune system to reduce the abundance of bacteria in the MAM7 treated group as shown by the reduction of daily bioluminescence measurements.

Alternatively, the control bead groups treated animals demonstrated daily increases of bacterial abundance for five days post infection. Similar amounts of bacterial growth were noted for both the Gram-positive and Gram-negative species tested in this model. By the end of the experiment many of the bioluminescent values for individual animals in the MAM7 inhibitor treatment groups were indistinguishable from the background levels (levels measured on uninfected skin were 1⋅10^6^ photons/sec or 9⋅10^5^ CFU on average). With this the level of bacteria in these individuals has completely resolved or was below the threshold for a clinically significant infection. Bacterial burdens in the control group persisted through the end of the experiment, where many of the animals had a notable abscess or purulent drainage at the wound site (Figure 2(a)). Foreign bodies following surgery are often colonized by bacteria and can lead to increased levels of infection [42]. However, both *S. aureus* and *P.aeruginosa* did not attach to the nanobeads (Figure 5) and bacterial burdens in infections without nanobeads were similar to infections with the control beads. This suggests that the decreased bacterial colonization in the MAM7 inhibitor treatment group is linked to inhibition of bacterial attachment to cells, and not the presence of a foreign substrate (i.e. control nanobeads).

Elevated levels of MMP2 and MMP-9 have been associated with wounds that have poor healing outcomes [43,44] and have been identified as potentially modifiable factors to accelerate wound healing [45,46]. Expression levels of MMP-2 and MMP-9 were not significantly different between MAM7 inhibitor-, control bead-, and sham treated incisions. Our data is consistent with previous *in vitro* [30] and *in vivo* [33] studies where MAM7 inhibitor did not alter wound healing. In addition to the reduction in bacterial abundance, histological examination demonstrated that treatment with MAM7 inhibitor or control beads did not affect wound closure compared to the sham incision. This is similar to what was demonstrated in the burn wound models with infections treated with MAM7[33]. In both the *P. aeruginosa* and *S. aureus* groups that were treated with control beads the incisional wounds failed to completely heal. Whereas those treated with MAM7 inhibitor beads the incision were fully healed at 9 days post injury. Given the abundance of bacteria in the control bead groups, it is likely that the persistent infection was the cause of the wounds inability to heal. Increased levels of bacterial burden are known to inhibit wound healing [47,48]. The MAM7 inhibitor treatment was able to sufficiently reduce the bacterial burden in the wound to allow the wound to heal within nine days of surgery.

Neutrophils are an important component of the immune system for host defense and migration toward sites of injury and inflammation. In the examination for the presence of neutrophils at the surgical incision, at nine days post injury neutrophils were notably absent in the uninfected treatment groups (Figure 4). Both the MAM7 inhibitor and the GST bead control groups were similar to the uninfected incisions that received a sham treatment. Fundamentally, the lack of neutrophils in these groups represent an absence of acute inflammation. In the animals that received a bacterial infection, the number of neutrophils were minimal in the MAM7 groups compared to the GST treatment. Neutrophils were present in the GST treated groups at the site of infection, which is indicative of neutrophil recruitment to an active bacterial infection. The minimal number of neutrophils noted in the infected and MAM7 inhibitor treated groups is concordant with the bioluminescent imaging data, demonstrating the significant reduction in bacterial levels for the MAM7 inhibitor treated groups. The differences noted between the abundance of neutrophils in the infected and GST control treated groups and the MAM7 inhibitor treated groups is also supportive of the hypothesis that blocking the initial attachment of bacteria allows for the host to more quickly remove the bacteria and resolve the infection.

Synthetic materials, including polymers, can be colonized by bacteria and are often implicated in device-associated infections. We tested whether functionalized microbeads would be prone to bacterial colonization, imaging beads and bacteria used in the surgical site infection model following co-incubation. No bacterial adhesion to the bead surface was observed following up to 48 hours of co-incubation (Figure 5). Additionally, infections treated with GST control beads did not result in a higher bacterial burden relative to untreated infections where no beads were introduced into the incision (Figures 1(d) and 2(d)).

Taken together, the current study demonstrates that the use of GST-MAM7 beads as a bacterial adhesion inhibitor effectively reduces colonization by clinically relevant bacterial pathogens in an *in vivo* model of surgical site infection. In contrast to a previous study, where the MAM7-based inhibitor showed efficacy against *P. aeruginosa* in a burn wound infection model, and was administered daily, the present study is based on administration of a single dose of the inhibitor prior to wound suture. Additionally, the present study shows, for the first time, that the MAM7-based adhesion inhibitor has broad-spectrum efficacy, decreasing bacterial loads in both a Gram-negative and a Gram-positive infection to or below the detection limit. In conclusion, use of an inhibitor that blocks the initial adhesion of bacteria to tissues is a promising approach to preventing SSIs and may be able to reduce bacterial infection rates and, thus, support antibiotic sparing strategies in an era of increasing challenges due to antibiotic resistance.
